# A single-center’s uric acid profile in girls with Turner syndrome

**DOI:** 10.3389/fendo.2024.1442166

**Published:** 2024-11-22

**Authors:** Song Guo, Qiuli Chen, Jun Zhang, Meihua Wei, Rujiang Zheng, Bing Wang, Yanhong Li, Huamei Ma, Xiaoyun Jiang

**Affiliations:** ^1^ Department of Pediatrics, First Affiliated Hospital, Sun Yat-sen University, Guangzhou, Guangdong, China; ^2^ Department of Child Healthcare, Shunde Women and Children’s Hospital, Guangdong Medical University, Foshan, China

**Keywords:** Turner syndrome, serum uric acid, lipids, insulin resistance, stanozolol

## Abstract

**Background:**

Metabolic disorders are common in individuals with Turner syndrome (TS). Hyperuricemia is associated with metabolic syndrome. This study investigated the serum uric acid (SUA) profile in patients with TS.

**Methods:**

A retrospective observational study was conducted with 145 patients with TS. A total of 72 normal girls were in the control group from 2015 to 2024: 86 TS patients were treated with growth hormone (GH), 80 with stanozolol, and 52 with estrogen.

**Results:**

Hyperuricemia was present in 33.1% (47/145) of patients with untreated TS and in 16.67% (12/72) of the controls (*P* < 0.001). Multivariable linear regression analysis showed that BMISDS, fasting serum glucose, and eGFR explained 34.4% (model *R* ^2^ = 0.344) of the total variation in SUA in the untreated TS group. SUA and SUASDS (SUA standard deviation score) levels generally showed a slow rising tendency with age. SUA increased significantly in the first year of stanozolol initiation (*P* = 0.032), while adding estrogen and stanozolol improved the lipid profile during the whole assessment period.

**Conclusion:**

Girls with TS showed a slow rising tendency in SUA and SUASDS with age and had higher SUA and SUASDS levels and incidence of hyperuricemia compared to their healthy female peers. The independent risk factors for hyperuricemia in pediatric patients with TS were BMISDS, HOMA-IR, glucose, and eGFR. The incidence of hyperuricemia increased in the first year of stanozolol treatment.

## 1 Introduction

Turner syndrome (TS) affects 25–50 per 100,000 female individuals, which involves multiple organs through all stages of life and is caused by a full or partial deletion of the second X chromosome. The primary problems during childhood and adolescence are short stature and premature ovarian failure, which require growth promotion and sex hormone replacement treatment. Patients with TS are also at an increased risk of obesity, dyslipidemia, metabolic syndrome, and impaired glucose tolerance (1, 2). At the same time, an elevated cardiovascular risk with an atherogenic lipid and carbohydrate profile and impaired endothelial function might occur in their youth (3), which could be mitigated with growth hormone (GH) therapy (4, 5).

Uric acid is the end-product of purine metabolism in humans, and serum uric acid (SUA) differs according to age and sex in childhood and adolescence (6, 7). Elevated serum uric acid level is associated with cardiovascular diseases such as hypertension, atrial fibrillation, chronic kidney disease, heart failure, coronary artery disease, and cardiovascular death (8). Many studies had also demonstrated an association between hyperuricemia and metabolic syndrome (9–11), and it has been well recognized by the 1970s (12) as a component of metabolic syndrome (13). In addition, male, obesity, diastolic blood pressure, and serum triglyceride concentrations were found to be associated with an increased risk of hyperuricemia in a Chinese population (14). As a condition associated with a high risk of renal and cardiovascular diseases and the special treatment of growth promotion and sex hormone replacement in patients with TS, SUA might differ from those of healthy peers. However, to our knowledge, the uric acid profile in individuals with TS was not recognized.

In this retrospective observational study, we analyzed the SUA profile in patients with TS before and after growth promotion and sex hormone replacement treatment.

## 2 Materials and methods

### 2.1 Patients

This study enrolled 145 girls (aged 2.75–22.00 years) with a definitively diagnosed TS based on karyotype and 72 prepubertal normal girls (aged 2.00–10.00 years) with a normal karyotype recruited from a tertiary hospital in China from 2015 to 2024. Patients with heart diseases, type 2 diabetes mellitus, resistant hypertension, or using drugs to lower urate were excluded, and those with abnormal thyroid function were all treated and were euthyroid during the study.

Growth promotion therapy with GH (0.33 mg/kg/week) at a mean age of 10.79 ± 3.42 years and an addition of stanozolol (ST, 20–35 μg/kg/day) when diagnosis occurred later than 10 years or older were implemented (15) in this study. Oral estradiol was applied for puberty induction according to guidelines (15) at a mean age of 16.27 ± 2.93 years, with the dose increasing over 2 to 3 years.

This study was approved by the First Affiliated Hospital, Sun Yat-sen University’s Institutional Ethics Committee.

### 2.2 Study design

In all patients, height and weight were measured by two doctors after training. Serum creatine (Scr), serum uric acid (SUA), serum uric acid standard deviation score (SUASDS) according to SUA reference values in normal Chinese children and adolescents (6, 7), fasting insulin (Ins, mIU/L), fasting glucose (Glu, mmol/L) and lipids (total cholesterol [TC], high-density cholesterol [HDL-c], low-density cholesterol [LDL-c], and triglycerides [TGs]), IGF-1 at the start of GH therapy, and regular evaluations were carried out during GH, stanozolol, and estrogen therapy or a combination of those treatments. The homeostasis model assessment of insulin resistance index (HOMA-IR) was calculated as fasting insulin × fasting glucose ÷ 22.5 (16). Scr was used in the calculation of the estimated glomerular filtration rate (eGFR) using the modified Schwartz equation (eGFR in mL/min/1.73 m^2^ = *k* × (height in cm/Scr mg/dL), where *k* = 0.413) (17). Hyperuricemia was defined as SUA higher than 360 µmol/L (6.0 mg/dL) (18–21). The control group was only evaluated once.

### 2.3 Statistical analysis

SPSS v27.0 and R software were used. Categorical variables were expressed as numbers and frequencies. For continuous variables, normally distributed data were presented as means ± SDs, and non-normally distributed data were shown as medians and interquartile ranges. *χ* ^2^ test, unpaired Student’s *t*-test, and Mann–Whitney *U*-test were applied to determine differences among groups. Correlations were evaluated using Spearman’s or Pearson correlation analysis. By entering SUA as the dependent variable, the independent effects of risk markers on SUA were estimated with stepwise linear regression analysis. The most recent SUA level in untreated TS patients and those collected during different therapies were collected to study the SUA profiles in populations with TS during childhood and adolescence. A *p*-value <0.05 was considered statistically significant.

## 3 Results

The karyotypes of 145 TS patients were as follows: monosomy (42.76%, 62/145, 45,X), mosaic (4.14%, 6/145, 45,XX/46,XX), variant (20.68%, 30/145, 15 with 46Xq10, five with 46,XiXq, one with 46,X,del(X)(p11), one with 46,X,del(X)(p12), one with 46,X,del(X)(p22.2q27), one with 46,XXp-, one with 45,Xi(X)q1021pstk+, one with 46,X,del(p11.4), one with 45,X,15pstsb+, one with 45Xpsuidic, one with 46,X,del(X)p11.2, 1 with 45,X,1qh), and mosaic with variant (32.41%, 47/145, 16 with 45,X/46Xq10, 10 with 45,X/47,XXX, eight with 46,Xr(X)/45,X, four with 45,X/46,X+mar, three with 46,Xder(X)(p11.2)/45X, one with 45,X/46,X-Xdel(X)(q22), one with 45X/46Xder(X)pter, one with 46,Xidic(X)(p22)/45X, one with 45,X/46,X,iv(X)(qwq.2q26), one with 45,X/46,Xadd(X)(q26), and one with 45,X/46,Xder(X)(q21)). Hyperuricemia was present in 33.1% (48/145) of patients with TS and in 16.67% (12/72) of the control group. The baseline characteristics of untreated TS children (all the included patients with TS had no breast development) and the control group (prepubertal normal girls) are shown in **Table 1**. A total of 26 patients of TS showed pubertal hair, while their SUA (335.33 ± 88.69 μmol/L vs. 341.24 ± 71.26 μmol/L, *P* = 0.376) and SUASDS (0.68 ± 1.51μmol/L vs. 0.57 ± 1.23 μmol/L, *P* = 0.375) was not significantly different with those of patients without pubertal hair growth. Renal abnormality was observed in 22 cases: 10 (41.67%) horseshoe kidney, three (12.5%) kidney duplication, two (8.33%) renal hypodysplasia, two (8.33%) hydronephrosis, one (4.17%) renal cysts, one (4.17%) ectopic kidney, two (8.33%) renal echogenicity enhancement, and one (4.17%) renal resistance index increase. We evaluated the eGFR, SUA, and SUASDS and classified them into group 1 (with renal morphological abnormality, *n* = 22) and group 2 (without, *n* = 123). The eGFR in group 1 was lower than that in group 2 (106.98 ± 17.70 mL/min/1.73 m^2^ vs. 115.70 ± 17.29 mL/min/1.73 m^2^, *P* = 0.015); SUA and SUASDS were shown to be not significantly different. No gout was diagnosed during the follow-up.

**Table 1:** Baseline characteristics of prepubertal patients with untreated TS and controls.

	TS (*n* = 145)	Control (*n* = 72)	*p*
Age (years)	11.64 ± 4.23	6.94 ± 2.10	**<0.001**
BMI (kg/m^2^)	17.9 (4.37)	14.23 (1.7)	**<0.001**
BMISDS	0.45 ± 1.26	-0.73 ± 0.85	**<0.001**
Ht (cm)	126.9 ± 11.26	112.1 ± 11.25	**<0.001**
eGFR (mL/min/1.73 m^2^)	115.07 ± 20.95	116.26 ± 20.49	0.433
SUA (μmol/L)	335.46 ± 85.58	281.59 ± 68.53	**<0.001**
SUASDS	0.69 ± 1.49	0.07 ± 1.05	**0.001**
HOMA-IR	1.41 ± 1.18	1.23 ± 1.21	0.32
Glucose (mmol/L)	4.57 ± 0.49	4.54 ± 0.40	0.469
Ins (mIU/L)	5.31(4.91)	4.80 (3.80)	0.41
TC (mmol/L)	4.78 ± 0.74	4.64 ± 0.89	0.21
TGs (mmol/L)	0.98± 0.60	0.86± 0.49	0.17
HDL (mmol/L)	1.49 ± 0.31	1.52 ± 0.41	0.60
LDL-c (mmol/L)	2.79 ± 0.76	2.67 ± 0.49	0.20

BMISDS, body mass index standard deviation score; HOMA-IR, homeostasis model assessment of insulin resistance index; TC, total cholesterol; HDL-c, high-density cholesterol; LDL-c, low-density cholesterol; TGs, triglycerides; TS, Turner syndrome; SUA, serum uric acid; SUASDS, serum uric acid standard deviation score.

The bold values mean statistical significance.

The risk factors for elevated SUA were analyzed. We found that BMI, BMISDS, eGFR, HOMA-IR, glucose, and TGs were associated with SUA (*r* = 0.34, 0.303, -0.319, -0.185, 0.202, -0.234, and 0.291; *P* < 0.001, <0.001, <0.001, = 0.024, = 0.029, and = 0.006, respectively) in the TS group, which was not found in the control group. After multicollinearity evaluation, BMI was not entered into the model. The multivariable correlates of SUA were BMISDS (9.94%), glucose (8.70%), HOMA-IR (5.10%), and eGFR (12.88%), which together explained 34.40% (model *R* ^2^ = 0.344) of the total variation in SUA in the TS group (see **Table 2**).

**Table 2:** Independent factors of hyperuricemia with multivariable linear regression.

	*β* ^c^	Partial *R* ^2^	*p*
BMISDS	21.613	0.099	**<0.001**
eGFR (mL/min/1.73 m^2^)	-2.094	0.1288	<**0.001**
HOMA-IR	23.876	0.051	**<0.001**
Glucose mmol/L	-56.128	0.0879	**<0.001**
Model *R* ^2^ = 0.344			

We analyzed the independent factors of hyperuricemia by multivariable linear regression in factors that we found to correlate with SUA.

*β* ^c^, 1 μmol/L change in serum uric acid per one unit change in the independent predictor.

SUA in the four groups of different karyotypes (monosomy group, mosaic group, variant group, and mosaic with variant group) showed no significant difference (*F* = 0.66, *P* = 0.578).

**Figure 1:**
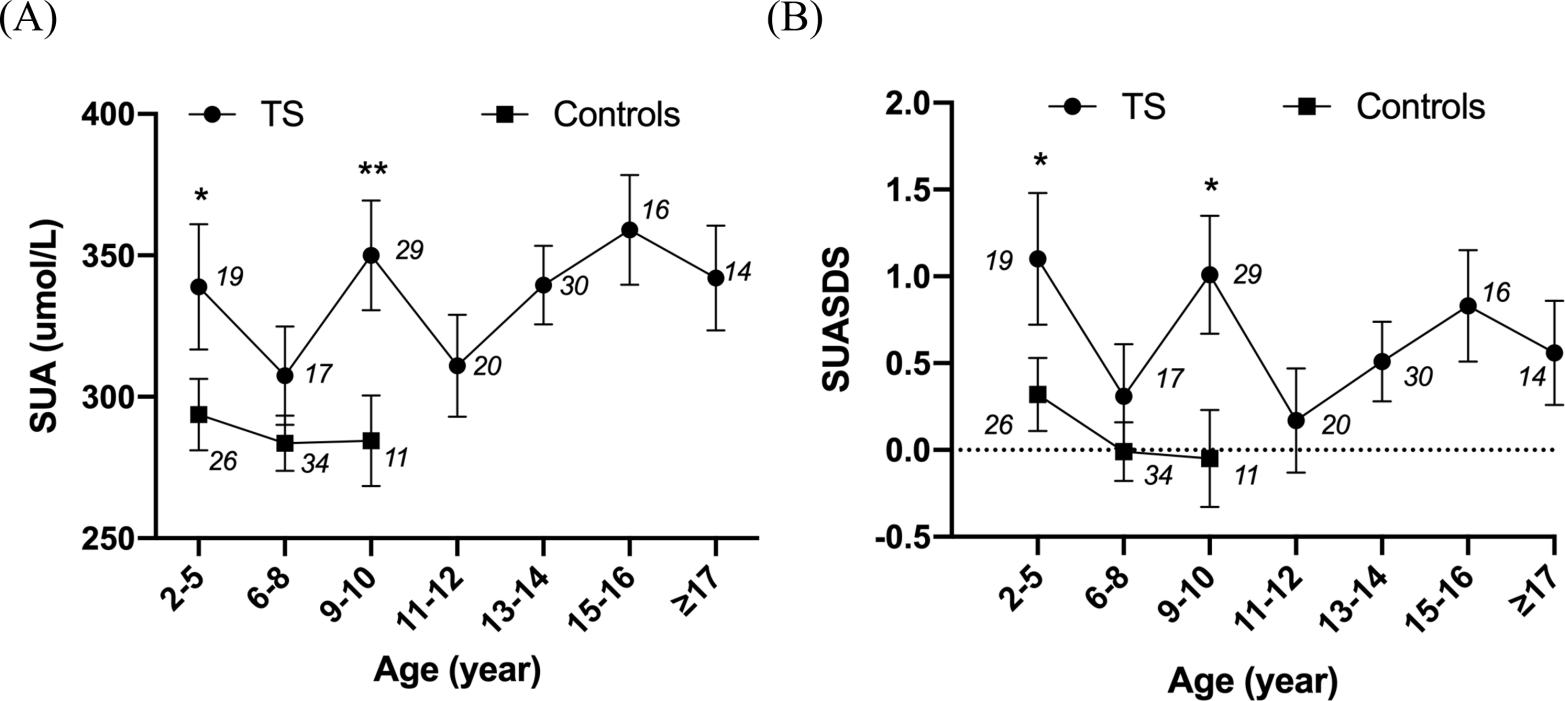
Line chart showing the relationship between SUA and age and SUASDS with age. *P<0.05, **P<0.01. The numbers in the figures are the number of cases in each age group. **(A)** Relationship of SUA level and age in patients with untreated TS and controls. **(B)** SUASDS level. 2–5 years: *N* = 19 (UA: 338.90 ± 96.62 μmol/L, SUASDS 1.10 ± 1.66), 6–8 years: *N* = 17 (UA: 307.48 ± 71.72 μmol/L, SUASDS 0.31 ± 1.20), 9 to 10 years: *N* = 29 (UA: 350.74 ± 106.56 μmol/L, SUASDS 1.01 ± 1.83), 11 to 12 years: *N* = 20 (UA: 311.05 ± 80.80 μmol/L, SUASDS 0.17 ± 0.35), 13 to 14 years: *N* = 30 (UA: 339.47 ± 79.01 μmol/L, SUASDS 0.51 ± 1.28), 15 to 16 years: *N* = 16 (UA: 359.00 ± 79.50 μmol/L, SUASDS 0.83 ± 1.26), ≥17 years: *N* = 14 (UA: 342.29 ± 69.54 μmol/L, SUASDS 0.56 ± 1.13) in patients with TS. 2–5 years: *N* = 26 (UA: 293.75 ± 64.28 μmol/L, SUASDS 0.32 ± 1.11), 6–8 years: *N* = 34 (UA: 283.56 ± 57.22 μmol/L, SUASDS -0.01 ± 1.00), 9 to 10 years: *N* = 11 (UA: 275.83 ± 60.72 μmol/L, SUASDS -0.22 ± 1.06), ≥11 years: *N* = 1 in controls. SUA and SUASDS levels were higher in patients with TS than those in controls at age of 2–5 years (*P* = 0.033, 0.033), 6–8 years (*P* = 0.101, 0.161), and 9 to 10 years (*P* = 0.003, 0.011).

Age was not linearly associated with SUA in patients with untreated TS. For those untreated patients with TS, the SUA and SUASDS levels showed a slow rising tendency with age, while in the control group, they showed a flat curve within the limit age range (see **Figure 1**).

As age affects SUA profiles, and we studied its changes with treatment of GH, stanozolol, or estrogen treatment after adjusting the patients’ age. No data from the empty control group was available due to the ethical reason of setting empty controls (GH treatment should start early) (22). We found that GH treatment (duration for the estrogen group and GH + estrogen group: 1.61 ± 0.79 vs. 1.16 ± 0.64 years, *P* = 0.11) did not affect SUA but decreased the insulin sensitivity (P_HOMA_ = 0.02, P_Ins_ = 0.014). Adding stanozolol to GH treatment (duration for GH group and GH + stanozolol group: 2.10 ± 1.10 vs. 1.16 ± 0.64 years, *P* = 0.103) did not affect SUA but decreased the serum glucose level (P_Glu_ = 0.024) and lipid profile (P_TC_ = 0.001, P_TGs_ = 0.032). Adding estrogen (duration for the GH group and GH + estrogen group: 1.66 ± 1.31 vs. 1.16 ± 0.64 years, *P* = 0.478) also improved TGs (P_TGs_ = 0.005) (see **Table 3**).

**Table 3:** Summary of SUA, glucose and lipid metabolism, and the effects of GH therapy, stanozolol, and estrogen in patients with TS.

	*N*		Age (years)	BMISDS	SUA (μmol/L)	SUASDS	Glucose (mmol/L)	Insulin (mIU/L)	HOMA-IR	TC (mmol/L)	TGs (mmol/L)	HDL-c (mmol/L)	LDL-c (mmol/L)
E	38	Before	15.46 (3.21)	0.87 (1.00)	382.90 (92.23)	1.24 (1.45)	4.34 (0.42)	11.41 (8.72)	2.33 (1.91)	4.50 (1.40)	1.15 (0.88)	1.61 (0.56)	2.75 (0.74)
		After	16.71 (1.62)	1.10 (1.12)	382.85 (68.59)	1.10 (1.56)	4.36 (0.54)	6.19 (4.62)	1.21 (0.96)	4.81 (0.74)	1.13 (0.76)	1.48 (0.25)	2.82 (0.60)
GH+E	19	Before	14.52 (4.50)	0.63 (1.24)	361.60 (90.79)	1.22 (2.28)	4.63 (0.55)	10.80 (5.36)	2.36 (1.28)	4.50 (0.78)	0.80 (0.28)	1.51 (0.49)	2.49 (0.39)
		After	15.91 (1.79)	0.45 (1.07)	391.88 (72.67)	1.52 (2.54)	4.30 (0.55)	9.65 (5.86)*	1.92 (1.27)*	4.36 (0.64)*	0.80 (0.27)	1.60 (0.23)	2.39 (0.49)*
GH	34	Before	12.09 (2.88)	0.41 (1.12)	332.87 (75.38)	0.55 (1.41)	4.59 (0.64)	6.1 (4.22)	1.33 (0.74)	4.84 (0.65)	0.93 (0.35)	1.48 (0.22)	2.91 (0.53)
		After	13.78 (2.87)	0.28 (1.10)	370.72 (104.7)	1.18 (1.51)	4.63 (0.75)	12.53 (9.48)	2.39 (1.50)	4.76 (0.62)	1.36 (0.99)	1.50 (0.32)	2.80 (0.43)
GH+ST	34	Before	11.17 (3.34)	0.92 (1.24)	347.96 (112.1)	0.92 (2.92)	4.59 (0.48)	5.50 (6.25)	1.02 (1.44)	4.67 (0.60)	1.07 (0.56)	1.44 (0.28)	2.84 (0.55)
		After	13.88 (2.52)	0.77 (1.18)	380.61 (99.29)	1.26 (2.75)	4.29 (0.46)*	13.98 (9.18)	2.78 (1.96)	4.20 (0.67)**	0.94 (0.44)*	1.24 (0.45)	2.58 (0.56)
GH	38	Before	13.62 (2.86)	0.28 (1.21)	331.75 (112.2)	0.56 (2.30)	4.58 (0.65)	9.61 (5.80)	2.11 (1.59)	4.94 (0.67)	0.88 (1.30)	1.42 (0.23)	3.07 (0.54)
		After	14.56 (2.72)	0.48 (1.16)	385.73 (109.6)	1.06 (2.05)	4.56 (0.48)	11.50 (6.82)	2.36 (1.44)	4.78 (0.60)	1.19 (0.55)	1.43 (0.27)	2.82 (0.53)
GH+E	19	Before	14.52 (4.50)	0.63 (1.24)	361.60 (90.79)	1.22 (2.28)	4.63 (0.55)	10.80 (5.36)	1.80 (0.95)	4.50 (0.78)	0.80 (0.28)	1.51 (0.49)*	2.49 (0.39)**
		After	15.91 (1.79)	0.45 (1.07)	391.88 (72.67)	1.52 (2.54)	4.30 (0.55)	9.65 (5.86)	1.92 (1.27)	4.36 (0.64)	0.80 (0.27)**	1.60 (0.23)**	2.38 (0.49)*

GH treatment (group with E vs. GH+E) improved the lipid profile (P_TC_ = 0.023, P_LDL-c_ = 0.011) and decreased the insulin sensitivity (P_HOMA_ = 0.02, P_Insulin_ = 0.014). Adding stanozolol (group with GH vs. GH + ST) decreased the serum glucose concentration (*P* = 0.024) and lipid profile (P_TC_ = 0.001, P_TGs_ = 0.032). Adding estrogen (group with GH vs. GH+E) decreased the TGs (*P* = 0.005).

BMISDS, body mass index standard deviation score; E, estrogen; GH, growth hormone; HOMA-IR, homeostasis model assessment of insulin resistance index; TC, total cholesterol; HDL-c, high-density cholesterol; LDL-c, low-density cholesterol; TGs, triglycerides; TS, Turner syndrome; SUA, serum uric acid; SUASDS, serum uric acid standard deviation score; ST, stanozolol.

*<0.05; **<0.01.

* :data of Before compared with data of Before, and data of After compared to data of After.

We retrospectively studied SUA in age-adjusted patients between groups treated with GH and treated with GH combined with stanozolol during the follow-up period. The results showed that the incidence of hyperuricemia was only higher at 1 year after combining stanozolol (see **Table 4**).

**Table 4:** SUA profile and incidence of hyperuricemia in age-matched girls with TS before and after stanozolol.

Group	0 year		0.5 year		1 year		2 years	
	GH	GH+ST	GH	GH+ST	GH	GH+ST	GH	GH+ST
*N*	24	24	24	24	24	24	14	14
SUA (μmol/L)	340.12 (93.81)	339.87 (80.45)	349.33 (104.53)	376.56 (100.09)	359.00 (100.86)	404.86 (115.40)	414.00 (81.13)	353.50 (90.27)
SUASDS	0.58 (1.91)	0.95 (2.14)	0.60 (1.21)	0.69 (1.85)	0.71 (1.38)	1.33 (2.47)	1.57 (2.28)	1.43 (3.02)
Hyperuricemia	10	7	9	14	9	16	12	11
*X* ^2^	0.504		2.087		4.608		0.243	
*P*	0.478		0.149		0.032*		0.622	

The incidence of hyperuricemia was higher since 0.5 year, significantly increased at 1 year, and was comparable to the group with only GH at the 2nd year of follow-up.

GH, growth hormone; TS, Turner syndrome; SUA, serum uric acid; SUASDS, serum uric acid standard deviation score; ST, stanozolol.

*<0.05.

## 4 Discussion

A range of cardiometabolic risk factors, such as obesity, impaired glucose sensitivity, and dyslipidemia, occur since childhood in individuals with TS and are associated with the risk of atherosclerosis and cardiovascular disease in adulthood (23, 24). Hyperuricemia, as one of those risk factors, is unclear in TS individuals. This study uncovered the uric acid profile in TS individuals, and BMISDS, HOMA-IR, fasting serum glucose, and eGFR were the independent risk factors of hyperuricemia. Growth promotion treatment with GH or ST and estrogen induction therapy did not affect the SUA level except that ST transiently increased the morbidity of hyperuricemia.

SUA was significantly higher in patients with TS than that in normal female peers. Based on the definition of hyperuricemia as an SUA level >6.0 mg/dL, the incidence of hyperuricemia was 33.1% in patients with TS, which was higher than that in normal female peers (16.67%). The incidence of hyperuricemia was 10.0% in a study of Chinese adults (9) and significantly higher in boys (2.7%) than in girls (1.9%) in preadolescent children from a Japanese study (20). The overall prevalence of SUA ≥310 μmol/L (5.16 mg/dL) among children was 10.1% in a study from China in children aged 3 to 6 years (25). Recent research described a mild elevation of SUA with age in normal Chinese girls, and the age-specific mean and upper reference value (97.5th percentile) slightly increased from 275 μmol/L (4.6 mg/dL) and ≤407 μmol/L (6.8 mg/dL) at 2 to 5 years to 308 μmol/L (5.2 mg/dL) and ≤432 μmol/L (7.3 mg/dL) over 14 years, respectively (7). The SUA in TS also showed a slow rising tendency with age. Given the narrow age range in controls, we compared to the age-matched normal girls from the above-mentioned study, and the mean value in TS patients over 10 years was also higher. We did not observe a change in SUA in patients with different karyotypes, suggesting that karyotypes did not affect SUA in girls with TS. To the best of our knowledge, this is the first study showing the SUA profile in patients with TS.

In normal karyotypes of children or adult populations, SUA is strongly associated with obesity, hypertension, high LDL-c, TC, and TGs (20, 26). Our study indicated that BMISDS, HOMA-IR, glucose, and eGFR were independent risk factors for hyperuricemia in patients with TS. Similar to those of previous studies in that BMI is associated with hyperuricemia (27, 28), BMISDS in patients with TS was also higher than that in control, and overweight or obese patients were more likely to have higher uric acid levels. This suggested that overproduction of UA was related to the accumulation of fat in the body of TS. Insulin resistance is an important determinant in the association between hyperuricemia and metabolic syndrome as both fructose-dependent and fructose-independent models that may mediate hyperuricemia with insulin resistance, fatty liver, and dyslipidemia can lead to hyperuricemia by reducing the kidney’s ability to excrete urate (29). Serum uric acid could mediate insulin resistance through the development of mitochondrial oxidative stress in endothelial cells (30). A previous study found that impaired glucose tolerance started in patients with TS since childhood, and the prevalence increased during adolescence, young adulthood, and early adulthood (10%, 16.7%, 21.4%, and 41.2%, respectively) (31). Ibarra-Gasparini D et al. also observed that early insufficient insulin secretion characterizes diabetes mellitus in TS. Interestingly, this study found that glucose level was negatively associated with SUA, of which the mechanism was unclear yet. It is worth noting that the glucose levels in this study were all within normal range, but it could not rule out the possibility that a relatively higher glucose level within the normal range was resulting from a relatively lower insulin level, which is a growth factor in childhood, and it might be associated to malnutrition or emaciation, which are generally signs of a low level of serum uric acid (20, 32–35). Some pediatric patients with TS may have a lower eGFR, which was also found in our study and might affect urate excretion (36). Lastly, although the mechanism of increasing SUA in TS was not fully explored by this study and further research is encouraged to solve the chicken-and-egg dilemma, we identified possible treatment targets of hyperuricemia and insulin resistance in TS.

SUA differs according to age and sex, and children over 12 years old (adolescence) showed an increase in SUA, and girls presented a lower SUA than boys from adolescence to menopause due to the hypouricemic effect of estrogen (6, 37, 38). We found that SUA in patients with TS had an upward trend with increasing age. Our results showed that SUA increased in adolescence in the population without spontaneous puberty initiation and SUASDS increased in people with adolescent hair, although there was no statistically significant difference, indicating the possible reason that the age and sex differences in SUA might be associated with androgenic hormones (6, 39). Nevertheless, our result indicated that the androgenic hormones that affected SUA in female individuals were mainly from adrenal glands rather than ovaries since the puberty of the adrenal gland maximizes in the late teenage years (40). Estrogen could decrease SUA by increasing the renal clearance of uric acid (38). However, the addition of estrogen in our study did not decrease SUA; this might be due to the lack of a uniform dosage that mimics normal puberty, and uric acid clearance needs to be further studied in individuals with TS considering their high risk of renal diseases.

Although there was no significant change in SUA, increased insulin resistance after GH treatment and improved lipid profile with the addition of GH, stanozolol, and estrogen were observed in this study. Increased insulin resistance was also found in a previous study (41). Lipids included TC, and LDL-c decreased with the addition with GH, which was also found in previous studies (42–44). Xiong H et al. observed that stanozolol did not affect lipid metabolism, while TC and TGs were decreased with the addition of stanozolol in this study (45). What is more, Irzyniec TJ et al. showed that hormone replacement therapy (HRT) does not affect lipid metabolism in TS women (44). However, we found that TGs and LDL-c decreased while HDL-c increased with estrogen replacement. The difference among the study results could be caused by the different study populations, which still needs further study for possible mechanisms to be explored. As lipid profile was an important metabolic and cardiovascular risk profile for patients with TS, the study provided supporting evidence that timely treatment with GH and estrogen is beneficial to the metabolic profile in individuals with TS.

Stanozolol is an anabolic–androgenic steroid which increases muscle growth and protein metabolism (46). For growth promotion, stanozolol induced the differentiation of chondrocytes and promoted growth plate development through the activation of JNK/c-Jun/Sox9 signaling (47). SUA was higher in stanozolol-treated individuals than in those not treated with stanozolol, although the difference was not statistically significant, which might be due to the limited sample size. However, when we further studied SUA change at different time points, we found that hyperuricemia increased in the first year of stanozolol treatment, which was consistent with findings in animal experiments (48). However, there was no statistically significant difference in the incidence of hyperuricemia between the stanozolol group and the control group in the second year. It might be related to several factors, of which inevitable statistical bias should be the first to consider due to the limited sample size, leading to underrepresentation of the results. Furthermore, the maximum anabolic effect of stanozolol on girls with TS has not been elucidated, and we speculated that it might take place at approximately 1 year after treatment, which needs further studies in patients with TS. Taken together, attention on SUA is still needed when stanozolol is planned to be used.

### 4.1 Limitations

This study is a retrospective observational study and not enough to figure out the mechanism of increased SUA profile in patients with TS. The age range of controls was not appropriately matched to those of patients with TS because we only included prepubertal children and aimed to match the ovarian failure station of TS. In addition, this study had a lack of enough sample size of patients during both stanozolol and estrogen treatment and had no data about the periodicity of estrogen and progesterone replacement or in adult individuals with TS. This study also did not consider the interacting effects of parental education, diet, and/or environment. Above all, further studies are needed.

## 5 Conclusion

Girls with TS showed a slowly rising tendency in SUA and SUASDS with age and have increased SUA and SUASDS levels and a higher incidence of hyperuricemia compared to their healthy female peers. The independent risk factors for hyperuricemia in pediatric patients with TS were BMISDS, HOMA-IR, glucose, and eGFR. The incidence of hyperuricemia increased in the first year of stanozolol treatment.

## Data Availability

The raw data supporting the conclusions of this article will be made available by the authors, without undue reservation.
